# *Wolbachia* elevates host methyltransferase expression to block an RNA virus early during infection

**DOI:** 10.1371/journal.ppat.1006427

**Published:** 2017-06-15

**Authors:** Tamanash Bhattacharya, Irene L. G. Newton, Richard W. Hardy

**Affiliations:** Department of Biology, Indiana University, Bloomington, Indiana, United States of America; Monash University, AUSTRALIA

## Abstract

*Wolbachia pipientis* is an intracellular endosymbiont known to confer host resistance against RNA viruses in insects. However, the causal mechanism underlying this antiviral defense remains poorly understood. To this end, we have established a robust arthropod model system to study the tripartite interaction involving Sindbis virus and *Wolbachia* strain *w*Mel within its native host, *Drosophila melanogaster*. By leveraging the power of *Drosophila* genetics and a parallel, highly tractable *D*. *melanogaster* derived JW18 cell culture system, we determined that in addition to reducing infectious virus production, *Wolbachia* negatively influences Sindbis virus particle infectivity. This is further accompanied by reductions in viral transcript and protein levels. Interestingly, unchanged ratio of proteins to viral RNA copies suggest that *Wolbachia* likely does not influence the translational efficiency of viral transcripts. Additionally, expression analyses of candidate host genes revealed *D*. *melanogaster* methyltransferase gene *Mt2* as an induced host factor in the presence of *Wolbachia*. Further characterization of viral resistance in *Wolbachia*–infected flies lacking functional *Mt2* revealed partial recovery of virus titer relative to wild-type, accompanied by complete restoration of viral RNA and protein levels, suggesting that *Mt2* acts at the stage of viral genome replication. Finally, knockdown of *Mt2* in *Wolbachia* uninfected JW18 cells resulted in increased virus infectivity, thus demonstrating its previously unknown role as an antiviral factor against Sindbis virus. In conclusion, our findings provide evidence supporting the role of *Wolbachia*–modulated host factors towards RNA virus resistance in arthropods, alongside establishing *Mt2’s* novel antiviral function against Sindbis virus in *D*. *melanogaster*.

## Author summary

Effective vector control is critically important to reduce the incidence of diseases caused by arthropod transmitted viruses. One proposed strategy involves the use of endosymbiotic bacteria *Wolbachia pipientis* as a novel biocontrol agent to prevent RNA virus transmission in mosquitoes. Previous work in the field suggests that the presence of this bacterium induces virus resistance within the host. However, the underlying mechanism of this antiviral phenotype is poorly understood, impeding its widespread use. Using the alphavirus, Sindbis as our model, we explored the tripartite interaction between the virus and the endosymbiont within its natural host, the fruit fly *Drosophila melanogaster*. In this study, we show that *Wolbachia* negatively influences multiple important aspects of the virus life cycle, extending our current understanding of the molecular nature of this interaction. We also provide evidence highlighting the role of a host gene, *Mt2*, in *Wolbachia*–mediated antiviral resistance, while uncovering its previously unknown role as an antiviral host factor against Sindbis virus.

## Introduction

Heritable symbioses are pervasive in nature and exceedingly common in the insect world, where many endosymbiotic associations have been described [1]. *Wolbachia pipientis* is an alpha-proteobacterial, maternally transmitted endosymbiont that invades insect host populations by manipulating host reproduction, favoring infected females [2]. It is present in approximately 40% of insects, including several species of *Drosophila* and important disease vectors such as *Aedes albopictus* and *Culex* species of common house mosquitoes [3]. In *D*. *melanogaster*, *Wolbachia* strain *w*Mel exhibits weak reproductive manipulation [4], but recent work has shown that this *Wolbachia* strain can also protect *D*. *melanogaster* from common viral pathogens such as Drosophila C virus (DCV), cricket paralysis virus (CrPV), and Flock House virus (FHV), as evidenced by increased survival and delay in virus accumulation [5, 6]. Not surprisingly, *Wolbachia*–mediated antiviral protection, so-called “pathogen blocking” is considered an exciting phenomenon that could be leveraged to reduce arthropod-transmitted diseases [7].

While *A*. *albopictus* infected with its native *Wolbachia* strain (*w*AlbB) has little to no effect on RNA virus replication (such as Dengue), transinfection of non-native *Wolbachia* strains like *w*Mel into this mosquito species, as well as the naturally uninfected *Aedes aegypti*, have been shown to induce pronounced antiviral resistance [8, 9]. Moreover, field trials conducted as a part of the global Eliminate Dengue project have demonstrated that these transinfected *A*. *aegypti* mosquitos can invade native *A*. *aegypti* mosquito populations, persisting over the seasons [10, 11]. However, although *Wolbachia’s* pathogen-blocking ability is currently being implemented in vector control, we know little to nothing about the mechanism underlying *Wolbachia*-induced antiviral resistance. Mosquitos as a model suffer from a lack of widely accessible genetic tools, coupled with the added complexity of introducing *Wolbachia* transinfections. Conversely, the antiviral phenotype was originally characterized in *Drosophila* and the genetic tractability of this model organism makes it an ideal candidate platform for a mechanistic dissection of the tripartite interaction [5, 6]. To this end, we have leveraged the power of *Drosophila* genetics, combined with the molecular tractability of a parallel tissue culture system, to probe previously uncharacterized aspects of *Wolbachia*-mediated resistance against the prototype alphavirus, Sindbis virus (SINV). In this study, we show that presence of *Wolbachia* in *D*. *melanogaster* results in reduced SINV infectivity, viral RNA replication and protein synthesis. Furthermore, we have identified the DNA/RNA methyltransferase gene, *Mt2* as a potential host factor responsible for *Wolbachia*-mediated antiviral resistance. Based on these results, we conclude that resistance towards SINV occurs at an early stage of virus replication that further affects subsequent stages of the virus life cycle and show evidence supporting the hypothesis that *Wolbachia* modulates the expression of a host methyltransferase gene (*Mt2)* to target virus RNA synthesis.

## Results

### *Wolbachia* reduces SINV infectivity

**Fig 1:**
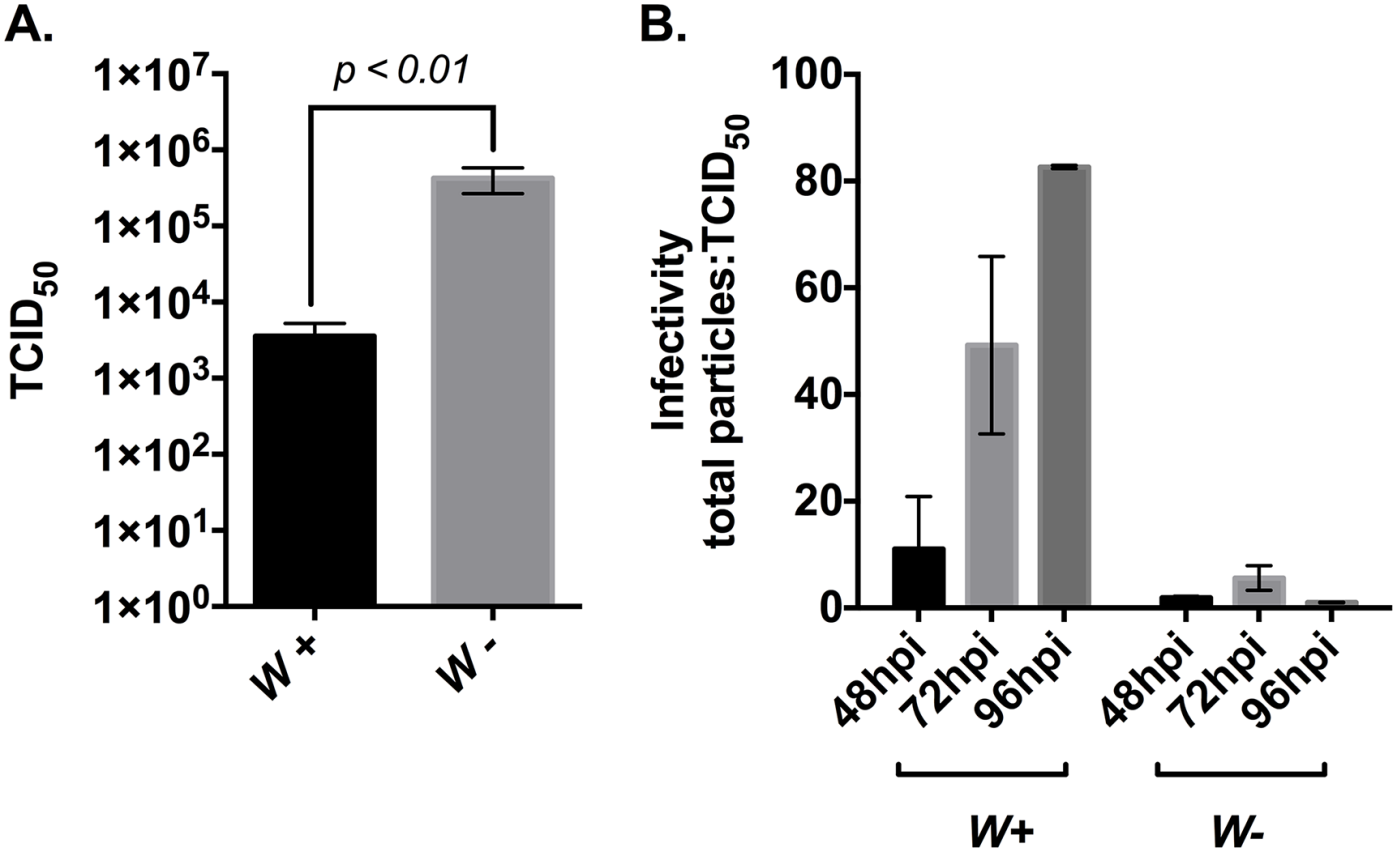
Effect of *Wolbachia* on SINV infectivity. (A) Presence of *Wolbachia* is correlated with reduced virus titer in *D*. *melanogaster* flies. Systemic viral infection was established in *Wolbachia*-infected (*left*) and *Wolbachia*-free (*right*) flies as described in Materials and Methods. Infection was allowed to last for 48-hours before whole fly tissues were harvested and assayed for the presence of infectious virus using end-point-dilution assay in BHK-21 cells. Numbers along the *y*-axis represent the dilutions that resulted in at least 50 percent cell death (TCID_50_). Data are mean values for three independent experiments. *P* values were calculated using Mann-Whitney U test (*p* = 0.0050) (B) Presence of *Wolbachia* in *D*. *melanogaster* derived JW18 cells lead to reduced virus particle infectivity over time. *Wolbachia*-infected (*left*) and *Wolbachia*-free (*right*) JW18 cells were infected with SINV at an MOI of 100 and media was collected at 48, 72 and 96 hours post infection. Samples were subsequently assayed for infectious virus using end-point-dilution assay and for particle numbers by qRT-PCR quantification of viral genome copies present in the media. Higher total particle: TCID_50_ numbers are indicative of lower virus particle infectivity. Values reported are the mean particle-to-TCID_50_ ratios of two independent replicates. All error bars represent standard error of mean (SEM).

We investigated the effect of *Wolbachia* on SINV titer in wild-type flies infected or uninfected with *Wolbachia* strain *w*Mel, which we refer to as *Wolb+* and *Wolb*- respectively from this point onwards (Fig 1A). We did not observe death as a consequence of virus infection in *Wolb+* or *Wolb*- flies. This was expected due to the non-pathogenic nature of SINV infection in *Drosophila*. Flies were collected 48 hours post-injection and virus titer was determined using end-point dilution assay on BHK cells. We found that infectious virus titer was significantly reduced in the *Wolb+* individuals compared to their *Wolb*- counterparts.

We and others have previously shown that SINV infectivity, measured by the ratio of total virus particles produced to infectious units, is influenced by the host cell environment in which the virus is cultivated [12]. To determine whether the presence of *Wolbachia* inside the host cell changes SINV infectivity, we used a cell culture based system comprised of *Drosophila melanogaster*-derived JW18 cell line infected with *Wolbachia* strain *w*Mel and an antibiotic-treated *Wolbachia* free control cell line, which we refer to as JW18-dox. Cells were infected with SINV at an MOI of 100 and samples of growth medium were taken at 48, 72 and 96 hours post infection (Fig 1B). We performed conventional end point dilution assay to quantify infectious particles released during infection and a previously described qRT-PCR based method to quantify the total copies of virus genome present in the growth medium over the course of the infection, which is equivalent to the total number of released virus particles [12].

We found that virus derived from *Wolb+* cells had a significantly high particle: infectious unit ratio relative to virus derived from *Wolb*- cells, indicating that on a particle basis this virus was less infectious than that derived from the *Wolb*- cells (Fig 1B). We have previously shown that SINV infectivity changes over the course of infection in both mammalian and mosquito cells, and also varies in a host cell dependent manner, i.e. virus grown in one cell line may be more infectious on a per particle basis than virus grown in a different cell line [12]. In the current study we found that infectivity of particles produced deteriorated over time during infection in the *Wolb+* cells.

These data show that not only does the presence of *Wolbachia* lead to a decrease in virus produced, but also a decrease the infectivity of the SINV particles produced during infection.

### SINV RNA synthesis is reduced in the presence of *Wolbachia*

During SINV infection, the incoming 49S genomic RNA functions as a mRNA to encode the viral replication complex and later acts as a template for the synthesis of minus-strand RNA. The minus strand RNA itself then serves as a template for the synthesis of nascent full length 49S genomic RNA and the transcription of smaller 26S sub genomic RNA, which encodes the viral structural proteins. Synthesis of these different viral RNA species and their subsequent translation is required for the formation of virus particles. Given that we found that the presence of *Wolbachia* resulted in fewer infectious virus progeny, we hypothesized that viral RNA synthesis is inhibited when *Wolbachia* is present at the time of SINV infection.

**Fig 2:**
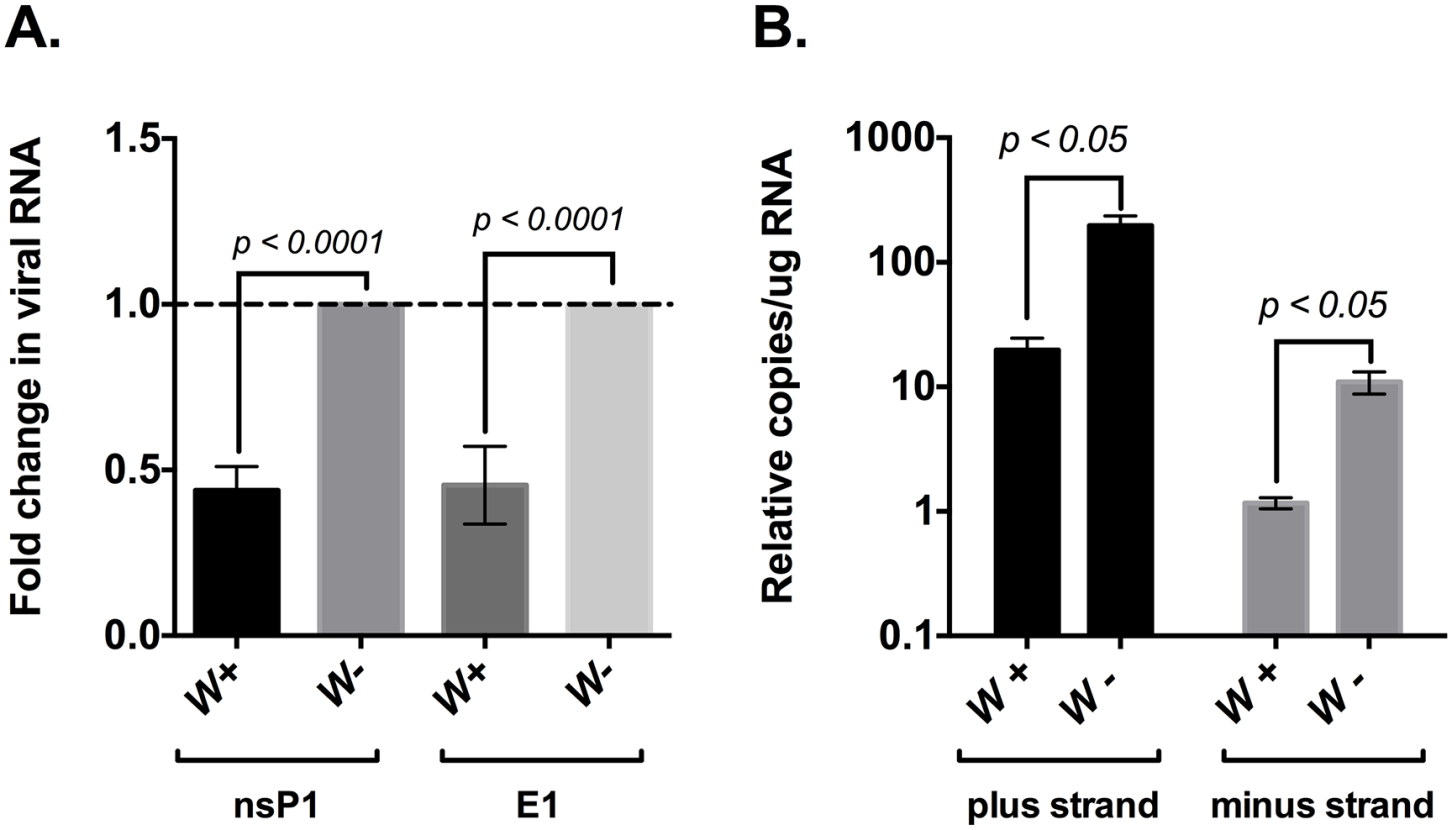
*Wolbachia* reduces SINV RNA synthesis. (A) Systemic virus infection was established in *Wolbachia*–infected (*left*) and *Wolbachia*–free (*right*) flies as described in Materials and Methods. Total RNA was isolated from fly tissue homogenates 48 hours post infection and assayed for fold change in RNA synthesis using qRT-PCR. Values reported are the mean of three independent biological replicates. *P* values were calculated using Mann-Whitney U test (nsP1: *p < 0*.*0001*, E1: *p < 0*.*0001*) (B) Absolute quantification of viral plus strand (*left*) and minus strand (*right*) RNA species observed during SINV infection in *Wolbachia*–infected and *Wolbachia*–free JW18 cells. Cells were infected with SINV at an MOI of 100 that lasted 96 hours, followed by extraction of total cellular RNA. Quantification of SINV RNA species was performed using qRT-PCR as described in Materials and Methods. Values were calculated using standard curves. *P* values were calculated using Unpaired t-test with Welch’s correction (plus strand: *p* = 0.043, t = 4.521, df = 2.063, minus strand: *p* = *0.046*, t = 4.457, df = 2.012). All error bars represent standard error of mean (SEM).

To test this, wild-type *Wolb+* and *Wolb*- flies were challenged with SINV. Infection was allowed to progress for 48 hours before the flies were collected and snap-frozen. Following tissue homogenization and RNA extraction, both relative, as well as absolute quantities of the viral RNAs were determined by qRT-PCR (Fig 2). We found that presence of *Wolbachia* is accompanied by 2.5-fold reductions in both viral genome (quantified using primers to nsP1 coding sequence) and subgenome (quantified using primers to E1 coding sequence) RNA levels, suggesting that viral RNA synthesis is inhibited in the presence of the endosymbiont (Fig 2A).

We subsequently repeated this experiment in our *D*. *melanogaster* derived cell culture system to further determine the absolute copies of plus strand and minus strand RNA produced during virus infection. JW18 and JW18-dox cells were infected with SINV at an MOI of 100. Infection was allowed to last for 96 hours before cells were collected for lysis and subsequent RNA extraction and absolute quantities of viral RNAs were determined using qRT-PCR (Fig 2B). In line with our previous observation in flies, we found an average 10-fold reduction in total copies of virus plus and minus strand RNA.

### Decrease in SINV protein expression in the presence of *Wolbachia* is a consequence of reduced virus RNA synthesis

**Fig 3:**
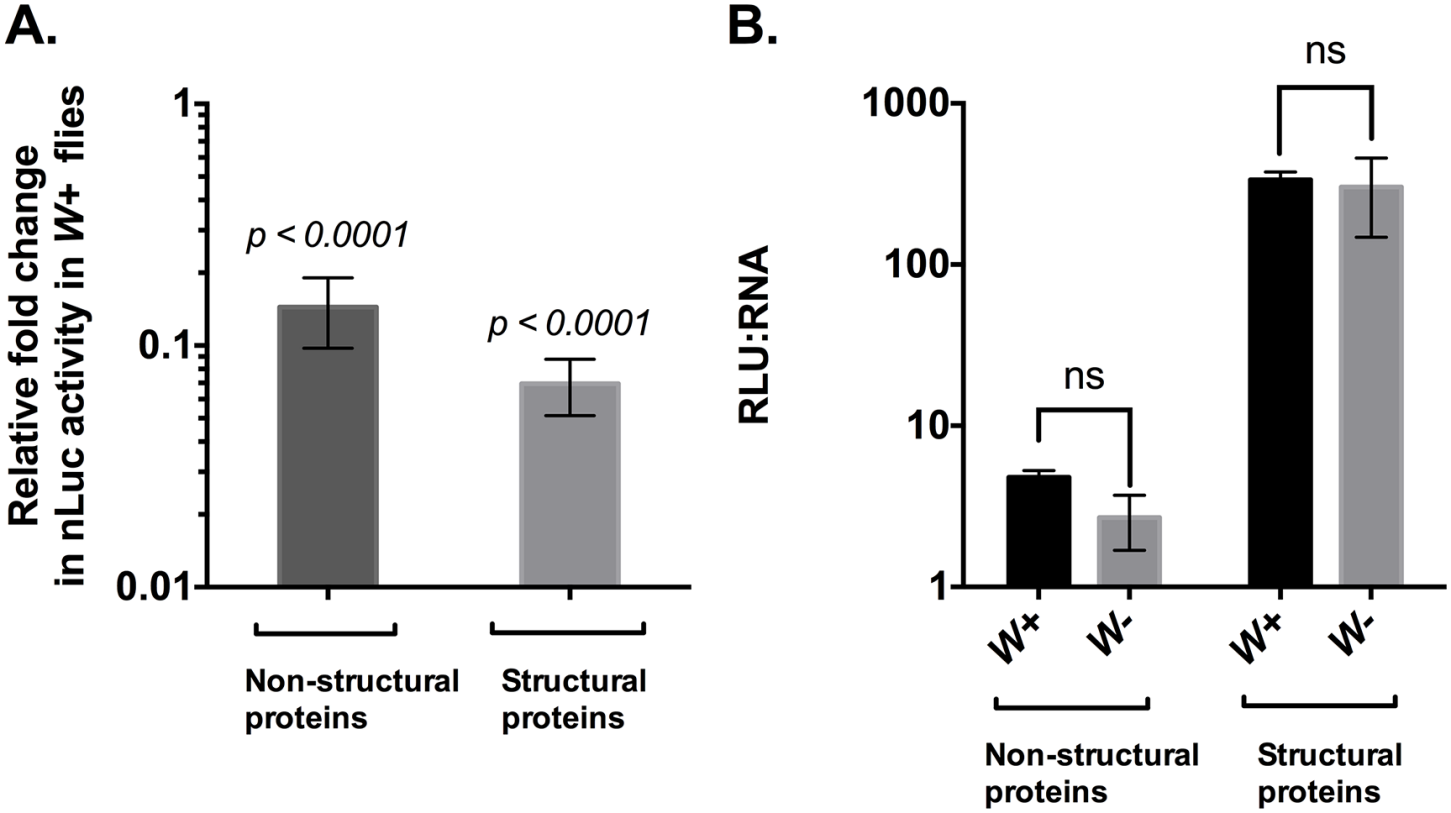
Viral protein synthesis is reduced in the presence of *Wolbachia* in flies. (A) *Wolbachia*–infected and *Wolbachia*–free flies were each infected with either SINV-nsP3-nLuc (*left*) or SINV cap-nLuc (*right*) viruses as described in Materials and Methods. Infection was allowed to last for 48 hours and fly tissues were harvested and assayed for luciferase activity to determine viral nonstructural (*left*) and structural (*right*) protein levels respectively. Reported values are relative to nLuc activity observed in *Wolbachia*–free (*W*-) flies, each set at a value of 1. Values represented the mean of three independent biological replicates. *P* values were calculated using Mann-Whitney U test (non-structural proteins: *p < 0*.*0001*, structural proteins: *p < 0*.*0001*) (B) Translation efficiency (RLU: RNA) of SINV non-structural (*left*) and structural (*right*) polyproteins was calculated as the ratio of total protein (shown above as the net luciferase output or RLU) to absolute copies of viral genomic and subgenomic transcripts, respectively. Absolute quantification of viral genomic and subgenomic RNA species was performed using qRT-PCR as described before. Values represented the mean of three independent biological replicates. *P* values were calculated using Mann-Whitney U test (non-structural proteins: *p = 0*.*20*, structural proteins: *p > 0*.*99*). All error bars represent standard error of mean (SEM).

Given our observed reduction in virus titer and RNA levels, we expected a concomitant reduction in virus protein synthesis. During infection, SINV non-structural proteins are translated as a polyprotein from the first open reading frame present in the 49S genomic RNA. Sometime later during infection, the 26S subgenomic RNA is translated as a polyprotein that subsequently gives rise to the virus structural proteins [13]. Therefore, expression of a luciferase reporter present within any non-structural or structural protein can be used as a proxy for the net translational activity from the genomic and subgenomic RNAs respectively. To this end, we used a luciferase based viral translation assay to quantitatively determine translation of SINV non-structural and structural proteins. We used SIN- nsP3-nLuc virus, in which nanoluciferase (nLuc) has been translationally fused to the hypervariable domain of SINV nsP3 protein, and SIN-cap-nLuc virus, which has nLuc translationally fused to the C-terminus of SINV capsid protein. Wild-type *Wolb+* and *Wolb*- flies were infected independently with each of the aforementioned viruses. Flies were collected 48hr post-infection and snap frozen and nLuc activity was measured post-homogenization (Fig 3A).

Our results indicated an average 10-fold reduction in nLuc activity in tissue derived from *Wolb+* individuals challenged with SIN-nsP3-nLuc virus, suggesting inhibition of SINV non-structural protein synthesis (Fig 3A). In contrast, quantification of nLuc activity in tissues derived from individuals challenged with SIN-cap-nLuc virus revealed a greater (~50-fold) *Wolbachia*–mediated reduction in nLuc activity (Fig 3A). These data indicate that *Wolbachia* causes a reduction in the synthesis of SINV non-structural and structural proteins and that expression of viral proteins synthesized off the subgenome are affected to a greater extent. However, it was not clear whether this was a consequence of decreased translational efficiency or simply a consequence of reduced quantities viral RNA available for translation (Fig 2).

To determine the cause of reduced viral protein synthesis, we extracted total RNA from the tissue homogenates of individuals infected with SIN-nsP3-nLuc or SIN-cap-nLuc viruses and determined absolute quantities of viral genomic and subgenomic RNAs using qRT-PCR as before. Following the calculation of viral protein, expressed in terms of luciferase activity (RLU), to viral RNA (copies/ug), we failed to find any significant difference in the protein-to-RNA ratios between *Wolbachia*–infected and—uninfected individuals, suggesting that viral protein translation is reduced due to scarcity of viral transcripts and that the smaller number of viral transcripts produced in the presence of the bacterium can be translated with regular efficiency (Fig 3B). Taken together, our data strongly indicates that *Wolbachia*-mediated inhibition of SINV infection occurs at an early stage of viral RNA replication that subsequently results in reduced viral protein synthesis and virus titer, which is consistent with previous reports regarding SFV infection in JW18 cells [14].

### Host Mt-2 is required for efficient *Wolbachia*-mediated inhibition of virus replication

Based on evidence from other systems, we investigated whether the antiviral resistance mediated by *Wolbachia* could be explained by modulation of host immune gene expression by the bacterium [15–19]. We tested this hypothesis by profiling the transcriptional activity of several candidate genes spanning different pathways that have been previously implicated as being a part of the host antiviral defense. The initial examination of the effect of *Wolbachia* on host gene expression was performed in the absence of a viral infection. We reasoned that it is important to consider the cellular environment that the virus is initially exposed to upon entry into the cell, not necessarily one that it encounters later during infection. In addition to canonical innate immune pathway components we examined the expression of *Mt2*, a gene encoding a nucleic acid methyl transferase previously shown to be required for DCV resistance in *Drosophila* [20]. Host gene expression was determined by qRT-PCR in *Drosophila* in the absence or presence of *Wolbachia*.

**Fig 4:**
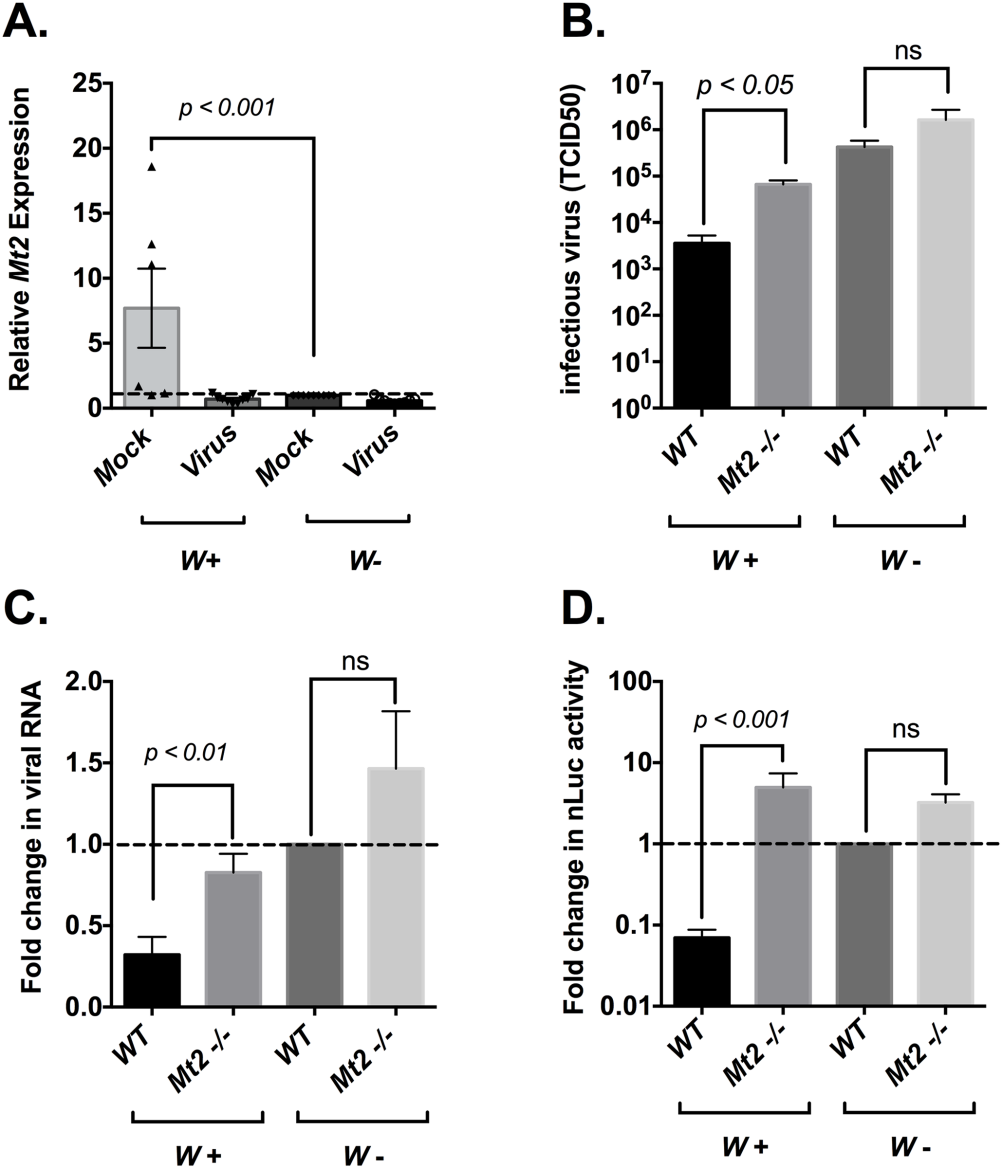
*Wolbachia* induced SINV resistance is dependent on host methyltransferase gene *Mt2*. (A) *Mt2* expression profile in *Wolbachia*–infected (*left*)and *Wolbachia*–free (*right*) flies either in the absence or presence of SINV infection. Virus infection was established as described in Materials and Methods. Infection was allowed to last for 48 hours followed by quantification of *Mt2* expression via qRT-PCR. *P* values were calculated using Mann-Whitney U test (*p < 0*.*001*) (B) Quantification of infectious virus produced during SINV infection in *Wolbachia*–infected (*left*)and *Wolbachia*–free (*right*) flies that are either wild-type (*WT*) or lacking functional *Mt2* gene (*Mt2 -/*-). Flies were challenged with SINV for 48 hours before whole fly tissues were harvested and assayed for the presence of infectious virus using end-point-dilution assay in BHK-21 cells. Values represent the mean of three independent biological replicates. *P* values were calculated using Mann-Whitney U tests between genotypes (*p < 0*.*05*) (C) Effect of *Mt2* loss on viral RNA synthesis in the presence of *Wolbachia*. SINV RNA was quantified using qRT-PCR by probing against the viral E1 gene. Values reported above are relative to viral RNA levels observed in *Wolbachia*–free wild-type flies. Values represent the mean of three independent biological replicates. *P* values were calculated using Mann-Whitney U tests between genotypes (*W+*: *p < 0*.*01*, *W*-: *p* = 0.71) (D) *Wolbachia*–infected (*left*)and *Wolbachia*–free (*right)* wild-type or *Mt2 -/*- flies were challenged with SINV cap-nLuc virus. After 48 hours post infection, fly tissues were harvested and assayed for luciferase activity to determine viral structural protein levels. Reported values are relative to nLuc activity observed in wild-type *Wolbachia*–free (*W*-) flies, set at 1. Values represent the mean of three independent biological replicates. *P* values were calculated using Mann-Whitney U tests between genotypes (*W+*: *p < 0*.*001*, *W*-: *p = 0*.*224*). All error bars represent standard error of mean (SEM).

We observed a general increase in expression of genes associated with canonical innate immune pathways such as Imd and Toll in the presence *of Wolbachia* (S1 Fig). Interestingly, we also observed a significant increase in the expression of the host methyl-transferase gene *Mt2*, with an average of 7-8-fold increase in transcript levels in the presence of *Wolbachia* (S1 Fig, Fig 4A). While a number of previous studies have discounted the role of canonical immune pathways in *Wolbachia*-induced pathogen blocking, the role of *Mt2* in this process is less clear. Interestingly, this elevated expression of *Mt2* in *Wolb+* flies decreased following SINV infection (Fig 4A).

To investigate the role of *Mt2* in *Wolbachia*–mediated inhibition of SINV infection, we looked at the effect of *Wolbachia* on virus infection in a previously characterized, homozygous loss-of-function mutant of *Mt2* (*Mt2 -/*-) [21]. SINV infection was established as before in *Wolbachia* infected and uninfected flies that were either wild-type or *Mt2 -/*- and the infection progressed for 48 hours before samples were collected, snap-frozen and virus titer was determined using end-point dilution assay on BHK cells (Fig 4B). SINV titer was, on average, 10-fold higher in *Wolb+ Mt2 -/*- mutants compared to *Wolb+* wild-type flies. Previous studies have established a direct correlation between *Wolbachia* density and the degree of antiviral resistance [22–24]. In light of this we examined whether the loss of function in the *Mt2* gene in the *Mt2 -/*- mutants is accompanied by a reduction in *Wolbachia* titer, which could potentially explain the observed loss in virus inhibition. Quantification of DNA collected from samples using qPCR showed no reduction in *Wolbachia* titer in the *Mt2 -/*- mutants compared to the wild-type, indicating that *Wolbachia* titer is not responsible for the observed reduction in virus inhibition (S2 Fig).

**Fig 5:**
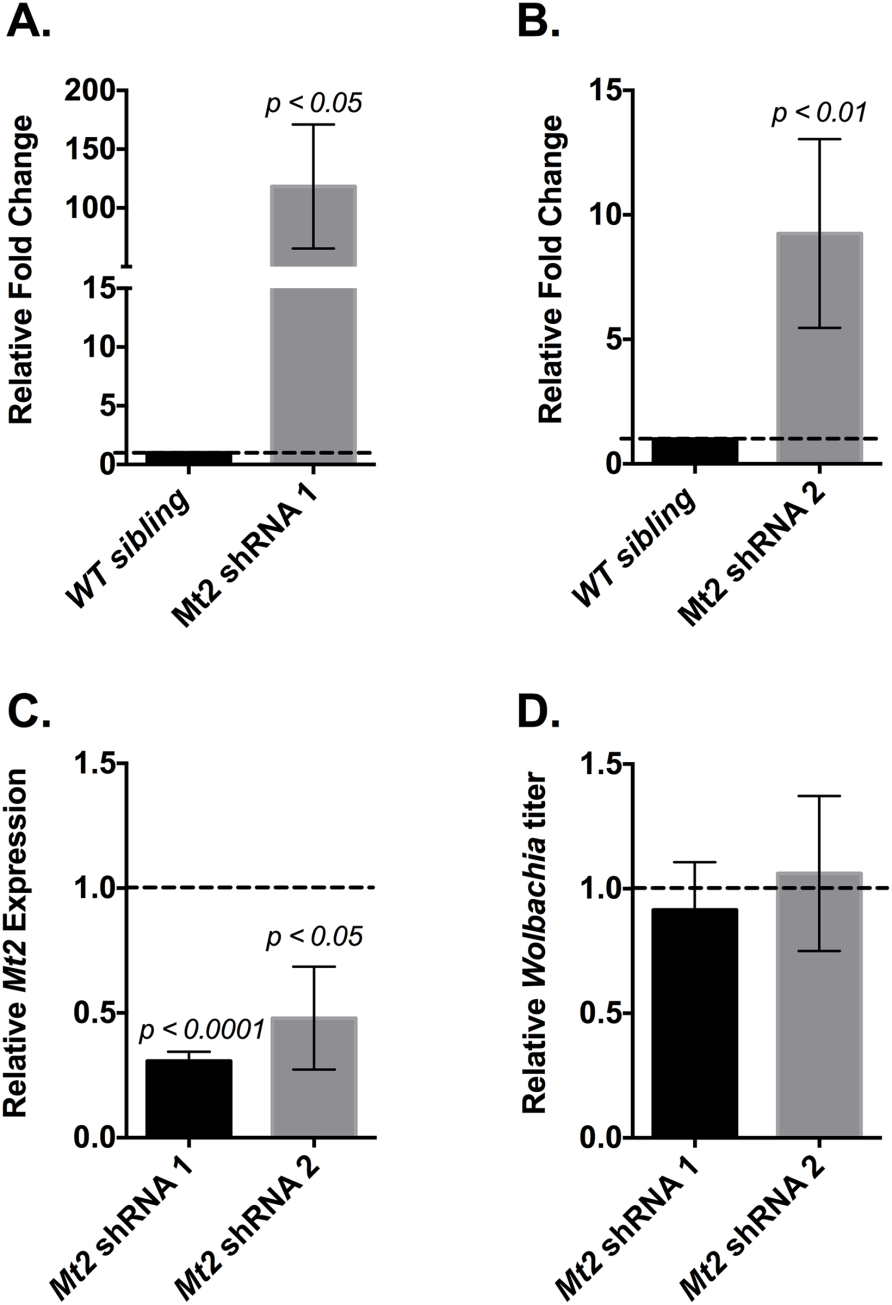
Effect of TRiP knockdown of *Mt2* expression in *Wolbachia* infected flies. *Mt2* expression was knocked down in *Wolbachia* infected transgenic RNAi fly stocks 38224 (*Mt2* shRNA 1) and 42906 (*Mt2* shRNA 2) by driving *Mt2* shRNA expression via chromosome II Act5C-Gal4 driver (y^1^ w*; P{Act5C-GAL4}25FO1/CyO) as described in Materials and Methods. For each set of crosses, siblings lacking the expression of *Mt2* targeting shRNA were used as the wild-type controls. (A, B) Flies were challenged with SINV as described previously whereby infection was allowed to last for 48 hours before whole fly tissues were collected and assayed for infectious virus using end-point-dilution assay on BHK-21 cells. Values represent the mean of six independent biological replicates. (C) Quantitative analyses of *Mt2* expression in TRiP mutant flies relative to their respective wild-type sibling controls (set at 1) was performed using qRT-PCR on total RNA extracted from fly tissues. Values for each column represent the mean of 6 and 3 independent biological replicates, respectively. *P* values were calculated using Mann-Whitney U tests (*Mt2* shRNA 1: *p < 0*.*0001*, *Mt2* shRNA 2: *p = 0*.*0361*). (D) *Wolbachia* titer was quantified using qPCR. Reported values are relative to respective wild-type sibling controls (set at 1) and are represented as the mean of two independent biological replicates. All error bars represent standard error of mean (SEM).

Similar results were obtained following shRNA-targeted knockdown of *Mt2* expression in two genetically distinct *Wolbachia*–infected fly lines, with each of the two shRNAs targeting a different area of the *Mt2* gene (Fig 5A and 5B, S3 Fig). Interestingly, the degree to which virus resistance was lost seemed to correlate roughly with the extent to which *Mt2* expression was reduced (Fig 5A–5C, S3 Fig).

Virus titer from *Wolbachia* uninfected wild-type flies were still found to be higher compared to the *Wolbachia* infected *Mt2 -/*- mutants (Fig 4B) although the data failed to meet the threshold for statistical significance. Additionally, comparison of virus titer from *Mt2-/*- flies with and without *Wolbachia* indicated that pathogen blocking is mediated by factors in addition to *Mt2*. However, once again the difference observed failed to reach statistical significance.

Given the fact that our initial characterization of *Wolbachia*–mediated SINV resistance showed viral inhibition occurred at an early stage of RNA synthesis and consequently resulted in reduced viral protein levels, we next sought to determine the effect of *Mt2* at these particular stages of virus replication. Quantification of SINV transcripts was performed using qRT-PCR as described above for absolute viral genome quantification (Fig 4C). Compared to *Wolbachia*–infected wild-type individuals, loss of *Mt2* resulted in an average of 2-3-fold increase in viral transcript levels. In contrast, we did not see any significant difference in the transcript levels between *Wolbachia* infected *Mt2 -/*- mutants compared to uninfected wild-type and *Mt2 -/*- mutants, showing that SINV RNA synthesis can be almost fully restored following the loss of *Mt2* function in the presence of *Wolbachia*.

While the data presented above indicated that inhibition at the stage of SINV RNA synthesis previously observed in the presence of *Wolbachia* is significantly reduced in *Mt2 -/*- mutant, it did not answer whether or not viral protein levels were restored in these mutants. We utilized our previously described SIN-cap-nLuc virus and luciferase based viral translation assay to quantify levels of SINV structural proteins in wild-type and *Mt2 -/*- mutants either in the presence or absence of *Wolbachia*. We found significantly higher expression of SINV structural proteins in the *Mt2 -/*- background (*Wolb+* and *Wolb*-), relative to that observed in their wild-type counterparts (Fig 4D). This result implies that the antiviral effect of *Mt2* targets a stage of viral RNA synthesis, with the loss of *Mt2* restoring the RNA and consequent protein levels to wild-type *Wolb*- levels.

We next examined whether the antiviral effect of *Mt2* against SINV could be independent of *Wolbachia*, as it has been reported before to be antiviral in the context of native DCV infection in *D*. *melanogaster* [20]. To test whether overexpression of the *Mt2* gene by itself resulted in SINV resistance, we used Gal4-UAS expression system to overexpress *Mt2* in *Wolbachia* uninfected flies. While we were only able to achieve modest levels of overexpression, following infection with SINV, *Mt2* overexpressing individuals were found to accumulate virus at an average of 2-fold lower titer than their wild-type sibling controls (S4A Fig). Levels of viral RNA in *Mt2* overexpressing flies were, on average, 2.5 fold lower compared to wild-type sibling controls (S4B Fig).

### Loss of *Mt2* leads to increased SINV particle infectivity in the absence of *Wolbachia*

To further investigate the antiviral role of *Mt2* against SINV, we asked whether *Mt2* is responsible for our initial observation regarding the reduction of virus particle infectivity in the presence of *Wolbachia*. To this end, we utilized our *Wolbachia* uninfected JW18-dox cell culture model to knock down expression of *Mt2* using targeted dsRNA against the methyltransferase gene. Cells were transfected with either *Mt2*-specific, or non-targeting dsRNA. SINV infection was established 48-hours post-transfection at an MOI of 100 and infection was allowed to last for 96 hours before cells and media were harvested separately.

**Fig 6:**
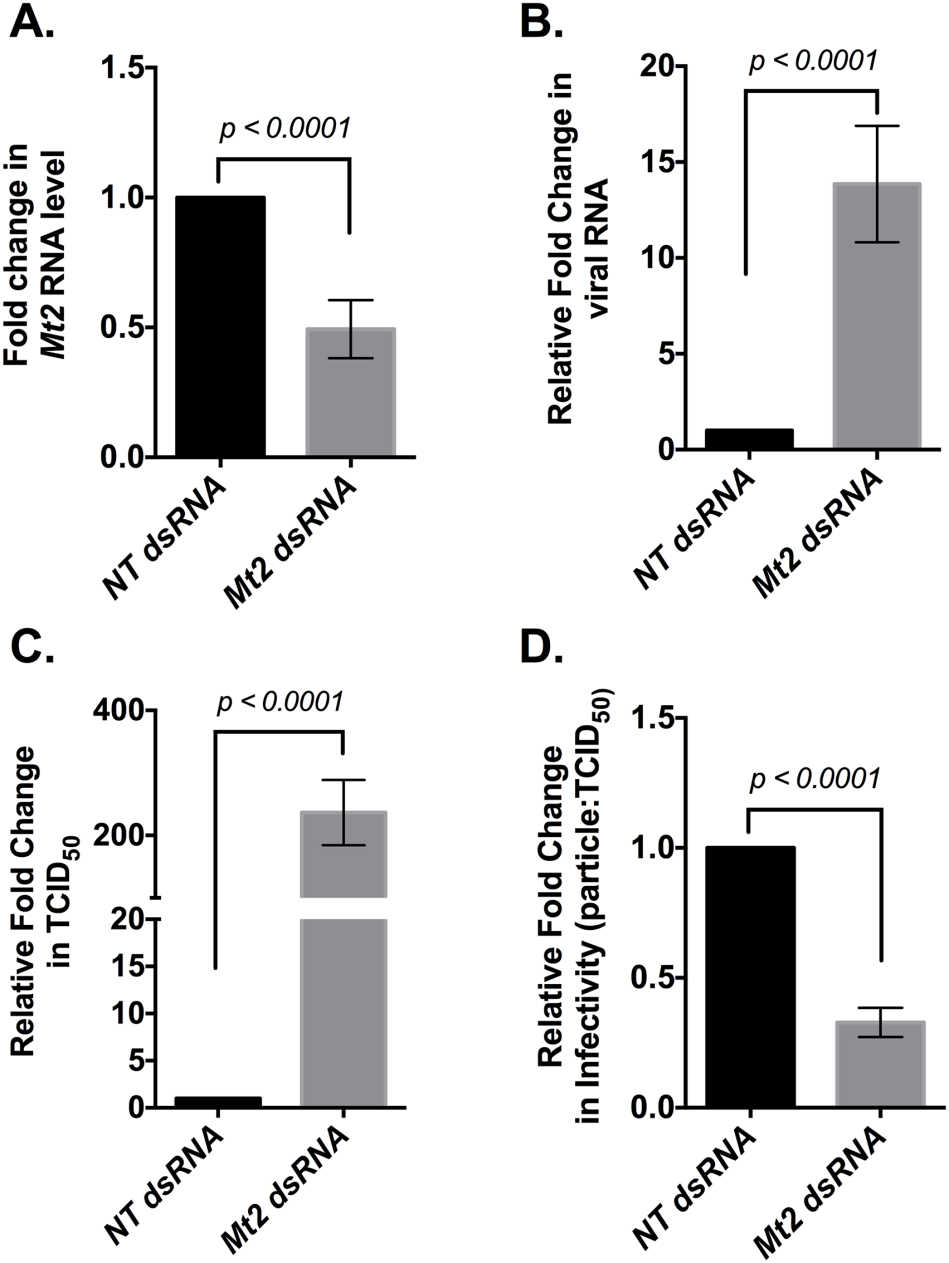
*Mt2* antiviral activity does not depend on the *Wolbachia* infection status of the host. *Mt2* expression was knocked down in D. *melanogaster* derived *Wolbachia*–free JW18 cells using dsRNA against the host methyltransferase gene. Approximately 48-hours post-transfection, cells were challenged with SINV at an MOI of 100 as described in Materials and Methods. Infection was allowed to last for 48 hours before harvesting the cells and media. Total cellular RNA was extracted from cells as described in Materials and Methods and assayed for host *Mt2* (A) and viral *E1* (B) gene expression. Values represent the mean of five independent biological replicates. *P* values were calculated using Mann-Whitney U tests (*Mt2*: *p < 0*.*0001*, *SINV E1*: *p < 0*.*0001*). (C) Media was collected 48 hours post infection and infectious virus particle was quantified as before, using end-point-dilution assay on BHK-21cells. Values represent the mean of five independent biological replicates. *P* values were calculated using Mann-Whitney U tests (TCID50: *p < 0*.*0001*). (D) Infectivity of virus particles produced was determined as described earlier, whereby total number of virus particles were quantified using qRT-PCR. As before, higher total particle: TCID50 numbers indicate poorer virus particle infectivity and relative reduction in particle: TCID50 ratio represents an overall increase in infectivity. Values represent the mean of five independent biological replicates. *P* values were calculated using Mann-Whitney U tests (particle: TCID50: *p < 0*.*0001*). All reported values are relative to non-targeting, control dsRNA treatments set at 1. All error bars represent standard error of mean (SEM).

Following RNA extraction from harvested cells, qRT-PCR based quantification of *Mt2* gene expression revealed an average 50% knockdown relative to non-targeting controls (Fig 6A). Probing the effect of *Mt2* knockdown on SINV RNA synthesis revealed viral RNA levels to be around 14-fold higher in cells transfected with *Mt2* dsRNA (Fig 6B), further confirming the results obtained in our animal model (Fig 4C).

Effect of *Mt2* knockdown on SINV particle infectivity in JW18-dox cells was performed as described previously by determining the ratio of total particles to the total number of infectious units. Virus titer was calculated using standard end-point dilution assay on BHK-21 cells. Relative fold change in virus titer from cells transfected with *Mt2* dsRNA was found to be on average, 200-fold higher compared to cells treated with non-specific dsRNA (Fig 6C). Consequently, virus particle infectivity was found to increase for virus derived from cells in which *Mt2* was knocked-down as evidenced by the 70 percent reduction in the particle-to-TCID_50_ ratio, indicating that *Mt2* plays a role in regulating virus particle infectivity (Fig 6D). Taken together, these results show *Mt2* to possess antiviral activity against SINV.

## Discussion

Arboviruses (arthropod-borne viruses) represent significant and imminent threats to public health across the globe due to the lack of commercially available vaccines or antiviral therapeutics. Several of the classic arboviral diseases are characterized by their long periods of absence, followed by sudden cases of re-emergence which may occur at unpredictable intervals, often decades apart [25, 26]. Therefore, until there are measures to inhibit viral replication and improve disease outcome, vector transmission control remains as the most effective strategy for preventing the spread of arboviral and other vector-borne pathogens. However, the increasing burden of diseases associated with dengue, chikungunya and Zika viruses reflect the ineffectiveness of traditional vector control strategies, thereby promoting the need towards developing unconventional, yet viable alternatives [27]. One promising approach involves harnessing the pathogen blocking properties exhibited by the maternally inherited endosymbiont *Wolbachia pipientis*.

At this time the causal mechanism behind *Wolbachia* induced RNA virus resistance in insects is poorly understood. Despite being observed in both native and transiently established *Wolbachia* infections [5, 6, 9–11, 14, 20, 22, 28–33], a considerable amount of phenotypic variability exists between different *Wolbachia* strains, hosts and viruses [22, 28–33]. In some cases, differences in titers of endogenous versus transinfected *Wolbachia* strains have been cited as a potential determining factor, while others have suggested a mechanistic divide between different host systems [8, 22]. However, it is important to note that despite these differences, antiviral resistance induced by *Wolbachia* is specific to RNA viruses, and this fact could help to narrow the underlying mechanism of virus inhibition. The data presented above demonstrate *Wolbachia’s* capacity to inhibit SINV replication in an arthropod host. We observed a significant decrease in the infectivity of SINV particles produced from *Wolbachia* infected cells. That is, while a significant number of virus particles were being produced in the presence of *Wolbachia* they were less infectious on a per particle basis than those produced in the absence of *Wolbachia*. This is important, as these results may help explain recent observations regarding the lack of detectable infectious virus particles present in salivary glands of *w*Mel-infected *A*. *aegypti* mosquitoes harboring other RNA viruses [31–33]. Finally, we also showed that this block happens early during infection at the level of viral RNA replication and that differential expression of the host methyltransferase gene, *Mt2*, plays a significant role in blocking SINV infection.

The physiology of the host cell harboring a virus influences the titer of the resulting virus progeny. Several factors, such as the stage of infection, for example, influence virus titer, whereby titer improves temporally as virus accumulates with the progress of infection. On the other hand, virus titer is also dependent on the physiological attributes of the cell from which the virus originates [12]. Given that the presence of *Wolbachia* induces resistance against different RNA viruses, it would be logical to think that the intracellular endosymbiont alters cellular physiology, creating an antiviral state that consequently influences the titer of the progeny virus produced. If this is indeed the case, where the induced antiviral effect is cell autonomous, then virus resistance would unconditionally require the presence of *Wolbachia* inside the cell. Furthermore, varying densities of *Wolbachia* in different tissues would then affect the degree of antiviral effect from cell to cell [34]. Indeed, *Wolbachia* is known to influence host gene expression [35, 36] and in certain cases, altered host gene expression negatively influences the development of malarial parasites in *Wolbachia*–infected vector species such as *A*. *aegypti* and *Anopheles gambiae* [15, 16]. In further support of an altered cellular environment upon *Wolbachia* infection, the bacterium was found to inhibit translation of SFV in JW18 cells as early as 7hpi with an absence of active transcriptional change in *Wolbachia* genes in response to virus infection [14]. This led to the conclusion that *Wolbachia’s* antiviral mechanism is either fast acting or is already present upon virus infection [14]. These results, and varying degrees of evidence, support the idea that *Wolbachia* might prime the host immune system, including factors such as the induction of ROS and alterations of host miRNA profiles, to induce antiviral resistance [17, 37 – 39]. However, several studies have discounted the role of canonical *Drosophila* innate immune components like RNAi as well as pathways such as Toll and Imd [19, 39]. In contrast, little attention has been given towards investigating the role of non-canonical host genes that may be involved in resistance against viruses. We identified the DNA/RNA methyltransferase *Mt2* as a host factor that restricted SINV infection in *Drosophila melanogaster* and established a link between its antiviral function and *Wolbachia*–mediated host resistance against SINV.

*D*. *melanogaster Mt2* gene encodes for a 40 kDa Dnmt2 protein which belongs to the most widely conserved Dnmt protein family and is the denoted as the lone cytosine methyltransferase present in many arthropods, including flies and mosquitoes [40, 41]. Its function is unconventional, given that Dnmt2 has been shown to methylate both DNA and tRNA^Asp^ at the cytosine-5 position (m^5^C). Dnmt2-directed cytosine methylation of genomic DNA in *D*. *melanogaster* is limited to specific loci on selected retrotransposons [42–44]. However, its robust RNA (tRNA) methyltransferase activity has been demonstrated both in vivo and in vitro, as evidenced by C38 methylation of tRNA^Asp^ and additional tRNAs [21, 45]. This is significant because Dnmt2 is required for DCV resistance in *Drosophila* and RNA immunoprecipitation experiments demonstrated interaction of Dnmt2 with DCV RNA during infection [20]. Here, we extend the antiviral role of Dnmt2 in SINV inhibition, both in the presence and absence of *Wolbachia*. Our results show that *Wolbachia* infection leads to an increase in *Mt2* expression prior to virus infection, thus the virus is being introduced to a system in which *Mt2* expression is high. Interestingly SINV infection led to a reduction of *Wolbachia*-induced *Mt2* expression. This is important to note as a prior study found no effect of *Wolbachia* on *Mt2* expression, but in that study the analysis of expression levels was performed after virus infection [22]. Additionally, we show that loss of *Mt2* results in a significant increase in SINV transcript levels as well as a concomitant increase in the synthesis of virus structural proteins and virus titer. We hypothesize that *Mt2* may function to inhibit viral RNA synthesis, which is in line with its previously described RNA binding properties.

Unlike traditional DNA methyltransferases, whose subcellular localizations are predominantly in the nucleus, evidence has been provided that Dnmt2 is distributed in the nucleus and the cytoplasm where SINV replication takes place. Indeed, fractionation studies of protein extracts from *Drosophila* embryos and localization of fluorescently tagged Dnmt2 in DCV infected fly tissues have confirmed the presence of Dnmt2 in the cytoplasm [44, 20]. The fact that cytoplasmic relocalization of Dnmt2 in response to cellular stress has been observed in other systems is significant, since elevated oxidative stress in the presence of *Wolbachia* is correlated with antiviral resistance in both flies and mosquitoes [37, 38, 46, 47]. In contrast to the data presented here Zhang et al. found that mosquito (*A*. *aegypti*) Dnmt2 promoted dengue virus replication [38]. It is not clear how to reconcile these contrasting observations, however it is not surprising that viruses from different viral families are affected differently by a host factor that is elevated in response to *Wolbachia*. Although further research is required to determine the exact nature of interaction between SINV RNA and Dnmt2, previous work involving DCV revealed significantly higher virus induced mortality in flies carrying a catalytically inactive mutant of Dnmt2, which suggests that enzymatic activity of Dnmt2 is required for its function as an antiviral factor [20]. Future work will focus on determining the RNA methylation status of the SINV genome in the presence or absence of *Wolbachia* and the relative importance of Dnmt2’s RNA methyltransferase activity in SINV inhibition.

Although we discovered a link between *Mt2* and *Wolbachia’s* pathogen blocking, the majority of the mechanistic details regarding this phenomenon remain unknown, and certainly other *Wolbachia* induced factors are likely to be involved. At this time, it is unknown whether Dnmt2 alters the methylation of SINV RNA. Given what is known about the functional implications of mRNA modifications in eukaryotic organisms, it is possible that the viral RNA acts as a target for cellular methyltransferases that subsequently influences the function of that RNA either positively negatively. Indeed, evidence of eukaryotic host methylation machinery acting as an either proviral or antiviral factor has been reported in the field [48–53]. Recent viral RNA methylation studies have found conserved, m^6^A modified regions spanning the RNA genomes of multiple members of *Flaviviridae* family, including Hepatitis C Virus (HCV), Zika Virus (ZIKV), Dengue Virus (DENV), Yellow Fever Virus (YFV) and West Nile Virus (WNV) [51, 52].

In the context of *Wolbachia* infection in insects, various degrees of evidence support the role of the endosymbiont in modulating the cytosine methylation profile of the host. In *D*. *melanogaster*, presence of native *Wolbachia w*Mel is correlated with increased methylation across all cytosine residues in male testes [54]. In addition, transcriptome-wide methylation profiling of virulent *w*MelPop infected *A*. *aegypti* mosquitoes reveal mostly random, yet widespread changes in the host cytosine methylation profile compared to *Wolbachia* uninfected individuals [55]. It is important to note that similar to *D*. *melanogaster*, mosquitoes such as *A*. *aegypti* and *A*. *gambiae* possess a single *Dnmt2* like cytosine methyltransferase [56, 57]. Therefore, given the selective nature of *Wolbachia* induced resistance towards viruses possessing RNA genomes, as well as growing evidence in the field that viral genome methylation regulates RNA virus infection, our data support the idea of *Wolbachia* induced modification of SINV RNA.

Whether or not *Wolbachia*-induced *Mt2* expression changes the methylation state of the viral RNA it is apparent that it has a biological effect on virus replication. Our data indicate this effect is exerted at an early time in infection and results in reduced levels of RNA synthesis. Future work fill focus on understanding how Dmnt2 interaction with viral RNA influences RNA function. The observation that infectious unit production is more severely inhibited than particle production *Wolbachia* infected hosts indicates that the genomes in the particles are less capable of initiating infection. It is known that methylation of RNA can change the stability of that RNA [48] which, if true for SINV RNA, could result in decreased viral RNA synthesis. Alternatively, *Wolbachia*-mediated Dmnt2 activity may lead to downstream alterations in the use of the genome as a template for translation and minus-strand RNA synthesis.

### Conclusions

Continued use of *Wolbachia* as an emerging vector control agent requires better understanding of the molecular mechanism underlying its antiviral resistance in the context of a tractable arthropod system. We provide evidence that the presence of *Wolbachia* reduces SINV particle infectivity and results in reduced virus titer. Furthermore, our data indicate a reduction in viral RNA synthesis, accompanied by decreased viral protein synthesis. Further characterization of early infection events is required to determine the exact nature of *Wolbachia*–mediated inhibition of SINV genome replication. Importantly, our data clearly demonstrate antiviral activity of the host RNA methyltransferase gene *Mt2* in the presence and absence of *Wolbachia*. Given the data provided in this study, it is likely that the effect of *Mt2* lies at the stage of viral RNA synthesis/stability. Future work will therefore focus on characterizing the nature of interaction between *Mt2* and SINV RNA. The fact that both *Wolbachia* mediated antiviral resistance and the antiviral effect of *Mt2* extends to members of different single stranded RNA virus families is significant, suggesting that existence of a common mechanism [5, 6, 9–11, 14, 20, 22].

## Materials and methods

### *Drosophila* husbandry, genetic crosses and virus injections

*Drosophila* fly stock 6326 (of a *W*^1118^ genetic background), carrying *Wolbachia* was obtained from the Bloomington Stock Center (BDSC), and used as the infected wild-type background. A corresponding *Wolbachia*–uninfected line was created through tetracycline treatment (approx. 20 ug/mL fed in the fly media for 3 generations). Wolbachia infection status was subsequently confirmed through quantitative PCR using published primer sets (S1 Table)[58]. The flies were repopulated with a wild-type microbiota post tetracycline treatment through culture in bottles previously occupied by untreated male flies of the same background (stock 6326). UAS-Mt2 and Mt2 loss of function flies (provided by S. Bordenstein) [54] were used to examine the role of the *Drosophila* DNA methyltransferase gene on the pathogen blocking phenotype. The Mt2 loss of function mutation is in a W^1118^ background as described by LePage et al. [54]. *Wolbachia*-infected TRiP mutant stocks 38224 (y^1^ sc* v^1^; P {TRiP.HMS01667} attP40) and 42906 (y^1^ sc* v^1^; P {TRiP.HMS02599} attP40) were used for shRNA-targeted knock-down of Mt2 gene expression by driving *Mt2* shRNA expression using previously described Act5C-Gal4 driver males (provided by Brian Calvi) y^1^ w*; P{Act5C-GAL4}25FO1/CyO, y^+^ (Fig 5) or y^1^ w*; P{w[Act5C-GAL4}17bFO1/TM6B, Tb^1^ (S3 Fig). For overexpression of Mt2, crosses were performed between UAS-Mt2 males and virgin Act5C-Gal4 driver females. Among the resulting progeny, straight-winged flies were considered to be overexpressing while siblings exhibiting Cyo phenotype were used as the wild-type control. All fly stocks were and maintained on standard cornmeal-agar medium supplemented with P/S at 25°C on a 24-hour light/dark cycle. In order to establish a systemic virus infection *in vivo*, flies were anesthetized with CO2 and injected in the thorax with 50nL of approximately 10^10^ PFU/mL of pelleted virus or control PBS using a glass capillary needle. Flies were collected two days post-infection, snap-frozen in liquid N_2_ and stored at -80°C for downstream processing.

In all stocks harboring *Wolbachia* infection, the *Wolbachia* strain was *wMel2*, confirmed by genotyping as shown in S5 Fig using primers described in Riegler et.al. 2005 [59] (S1 Table).

### Cell culture and infection of cells with SINV

*Wolbachia*-infected *Drosophila melanogaster* JW18 cells and corresponding doxycycline-treated *Wolbachia*-uninfected JW18_dox cells (a generous gift from W. Sullivan) were maintained at 24°C in Shields and Sang media (Sigma), supplemented with 10% heat-inactivated fetal bovine serum (FBS) and 1% Antibiotic-Antimycotic (Gibco). BHK-21 cells (American Type Culture Collection) were grown at 37°C under 5% CO_2_ in MEM (CellGro) supplemented with 1% L-Gln, 1% Antibiotic-Antimycotic (Gibco), 1% non-essential amino acids and 10% heat inactivated Fetal Bovine Serum (FBS). JW18 cells were seeded in a 6-well plate at a density of approximately 2.26x10^6^ cells/well 24h prior to infection. Infection was carried out using virus derived from BHK-21 cells, titered using a standard plaque assay. Virus was diluted in Shields and Sang media (Sigma) and the infection was established at an MOI = 100. For doxycycline cleared JW18-dox cells, around 50–55% infection was observed at 96 hpi. Mock infections were carried out by treating the cells equivalently, without the addition of virus.

### Viral translation assays

Quantification of viral genome and subgenome translation was performed by introducing SINV luciferase reporter viruses nsP3-nLuc and cap-nLuc, respectively into 2-day old virgin female flies as described. The samples were homogenized in 1X Cell Culture Lysis Reagent (Promega) after they were collected 2-days post infection and clarified via centrifugation at 16,000 × g for 2 min. The samples were then mixed with luciferase reagent (Promega), and luminescence was recorded using a Synergy H1 microplate reader (BioTech instruments). In all cases, the luciferase readings were normalized to the levels of SINV genomic and sub-genomic RNA present in the assayed samples, determined by using qRT-PCR methods with ΔΔCT calculation of transcript levels.

### *Wolbachia* density

*Wolbachia* density from fly homogenates and tissue culture cells were determined via qPCR on whole DNA using an Applied Biosystems StepOne Real-time PCR system (S1 Table) and SYBRGreen Chemistry (Applied Biosystems), previously described in [58]. *Wolbachia* density (and infection status) in JW18 cells was determined using DAPI staining, where *Wolbachia* were visualized in the form of cytoplasmic foci within the cells.

### *Mt2* knockdown in JW18-dox cells

Knockdown of *Mt2* expression was achieved in doxycycline treated JW18 cells using target dsRNA against the *Mt2* gene. *Mt2* dsRNA was synthesized from corresponding dsDNA generated using self-annealing primer sets. First, custom oligonucleotide was designed with a 5’ T7-Polymerase binding site (GAATTAATACGACTCACTATAG) followed by a 3’ target sequence specific to the *Mt2* coding region. Similarly, another oligonucleotide was designed with a similar 5’ T7 polymerase binding site followed by a 3’ end complementary to the 3’ end of the previous oligo (S1 Table). Polymerase chain reaction was carried out using 100uM of primers and Q5 High-fidelity Master Mix [NEB] to produce dsDNA. dsRNA was synthesized via in-vitro transcription of this dsDNA, using T7-RNA Polymerase in the presence of the 5'cap analog 7'G5'ppp5'G [New England Biolabs], followed by transfection of 500 ng dsRNA into JW18-dox cells using Lipofectamine LTX [Thermo Fisher Scientific]. Maximum knockdown was achieved at 48 hours post transfection.

### Real-time quantitative RT-PCR analysis

Whole flies were homogenized in TRIzol reagent, followed by RNA extraction. cDNA was synthesized using MMulV Reverse Transcriptase (New England Biolab) with random hexamer primers (Integrated DNA Technologies). Negative (no RT) controls were performed for each target. Quantitative RT-PCR analyses were performed using Brilliant III SYBR green QPCR master mix (Agilent) with gene-specific primers according to the manufacturer's protocol and with the Applied Bioscience StepOnePlus qRT-PCR machine (Life Technologies). The expression levels were normalized to the endogenous 18S rRNA expression using the delta-delta comparative threshold method (ΔΔCT). Fold changes were determined using the comparative threshold cycle (CT) method (S1 Table).

### Statistical analyses

*P-values* were calculated as described in the individual Fig legends. The average fold change (FC) in each experiment was calculated using the variable bootstrapping method, measuring the fold change between each potential pair of flies to determine the variability of the mean [12]. 95% confidence intervals (CI) were calculated using one sample t-test of log2FC values to determine the significance of distribution of the mean relative to the null using IBM SPSS Statistics Software 24 [60].

## Supporting information

S1 Fig Immune gene expression in the presence of *Wolbachia* infection in flies. Gene expression profile of candidate immune genes was examined in mock infected flies either in the presence or absence of *Wolbachia*. Total RNA was isolated from fly tissue homogenates 48 hours post infection and assayed for fold change in RNA synthesis using qRT-PCR as described in Materials and Methods. Primer sets used in the analyses can be found in the supplementary information (S1 Table). Values are relative to the respective transcript levels detected in *Wolbachia* free flies (set at 1) and represent the mean of three independent biological replicates. *P* values were calculated using Mann-Whitney U tests. All error bars represent standard mean of error (SEM).
(TIFF)

S2 Fig Loss of *Mt2* is correlated with increase in *Wolbachia* titer in flies. Relative *Wolbachia* titer in *Mt2 -/*- mutant flies was quantified using qPCR. Reported values are relative to isogenic wild-type flies (set at 1) and are represented as the mean of three independent biological replicates. All error bars represent standard error of mean (SEM).
(TIFF)

S3 Fig Effect of TRiP knockdown of *Mt2* expression in *Wolbachia* infected flies. *Mt2* expression was knocked down in *Wolbachia* infected transgenic RNAi fly stocks 38224 (*Mt2* shRNA 1) and 42906 (*Mt2* shRNA 2) by driving *Mt2* shRNA expression via chromosome III Act5C-Gal4 driver (y^1^ w*; P{w[Act5C-GAL4}17bFO1/TM6B, Tb^1^) as described in Materials and Methods. For each set of crosses, siblings lacking the expression of *Mt2* targeting shRNA were used as the wild-type controls. (A, B) Flies were challenged with SINV as described previously. Infection was allowed to last for 48 hours before RNA was extracted from whole fly tissues, followed by quantification of viral nsP1 and *Mt2* expression via qRT-PCR. Quantitative analyses of gene expression in TRiP mutant flies was performed relative to their respective wild-type sibling controls (set at 1). Values represent the mean of six and four independent biological replicates, respectively. *P* values were calculated using Mann-Whitney U tests (*Mt2* shRNA 1: nsP1 *p < 0*.*0001*, *Mt2 p < 0*.*0001* and *Mt2* shRNA 2: nsP1 *p < 0*.*01*, *Mt2 p < 0*.*01*). (C) *Wolbachia* titer was quantified using qPCR. Reported values are relative to respective wild-type sibling controls (set at 1) and are represented as the mean of three independent biological replicates. All error bars represent standard error of mean (SEM).
(TIF)

S4 Fig Effect of *Mt2* overexpression on SINV infection in flies. *Wolbachia*–free flies overexpressing *Mt2* was generated by crossing uninfected UAS-*Mt2* males with uninfected virgin Act5C-Gal4 driver females. Siblings generated through these crosses were used as wild-type controls. Flies were challenged with SINV that lasted for 48 hours before whole fly tissues were harvested. (A) Infectious virus was quantified as described previously, using end-point-dilution assay on BHK-21 cells. Values represent the mean of three independent biological replicates. *P* value was calculated using Mann-Whitney U test (*p* = 0.0476). (B) SINV RNA was quantified using qRT-PCR by probing against the viral E1 gene. Values represent the mean of three independent biological replicates. *P* value was calculated using Mann-Whitney U test (*p* < 0.001). All error bars represent standard error of mean (SEM).
(TIFF)

S5 Fig Genotyping of *Wolbachia* strains in *Mt2 -/*- flies. PCR based genotypic analyses was carried out to identify native *D*. *melanogaster Wolbachia* strain(s) present in the wild-type (*W*^*1118*^*)* and *Mt2 -/*- flies used in this study. (A) Genomic maps of common *Wolbachia* strains present in *D*. *melanogaster* indicate the location of distinct chromosomal IS5 transposon elements present or absent within either WD1310 or WD0516/7 loci. (B) Genomic region spanning the WD0516 loci was amplified using DNA isolated from *W*^*1118*^ and *Mt2 -/*- fly lysates (n = 3/sample) *(Left)*. Controls obtained from flies infected with *Wolbachia* strains either *w*Mel3, *w*Mel2, *w*MelCS2 or *w*MelCS *(Right)*. Increased shift in band migration indicate the presence of IS5 element at the WD0516 locus.
(TIF)

S1 Table Primer sets used in this study for qRT-PCR and PCR* analyses. (PDF)
