# Supplementary figures and images for: Wolbachia elevates host methyltransferase expression to block an RNA virus early during infection
Source: PLoS Pathog. 2017 Jun 15;13(6):e1006427. doi: 10.1371/journal.ppat.1006427 (PMC5472326; doi:10.1371/journal.ppat.1006427)

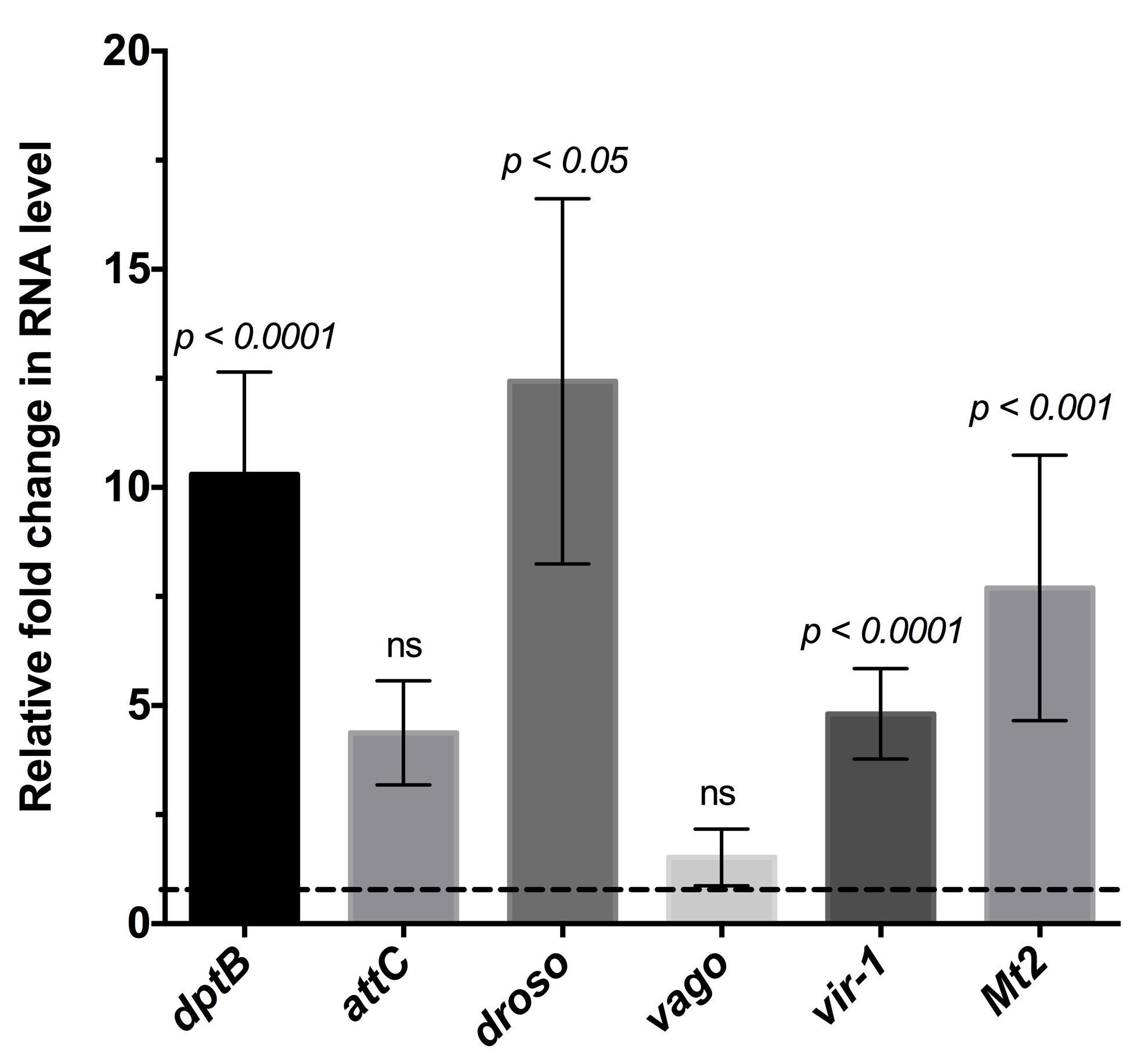

Supplement: S1 Fig — Gene expression profile of candidate immune genes was examined in mock infected flies either in the presence or absence of Wolbachia. Total RNA was isolated from fly tissue homogenates 48 hours post infection and assayed for fold change in RNA synthesis using qRT-PCR as described in Materials and Methods. Primer sets used in the analyses can be found in the supplementary information (S1 Table). Values are relative to the respective transcript levels detected in Wolbachia free flies (set at 1) and represent the mean of three independent biological replicates. P values were calculated using Mann-Whitney U tests. All error bars represent standard mean of error (SEM). (TIFF) [file ppat.1006427.s001.tiff]

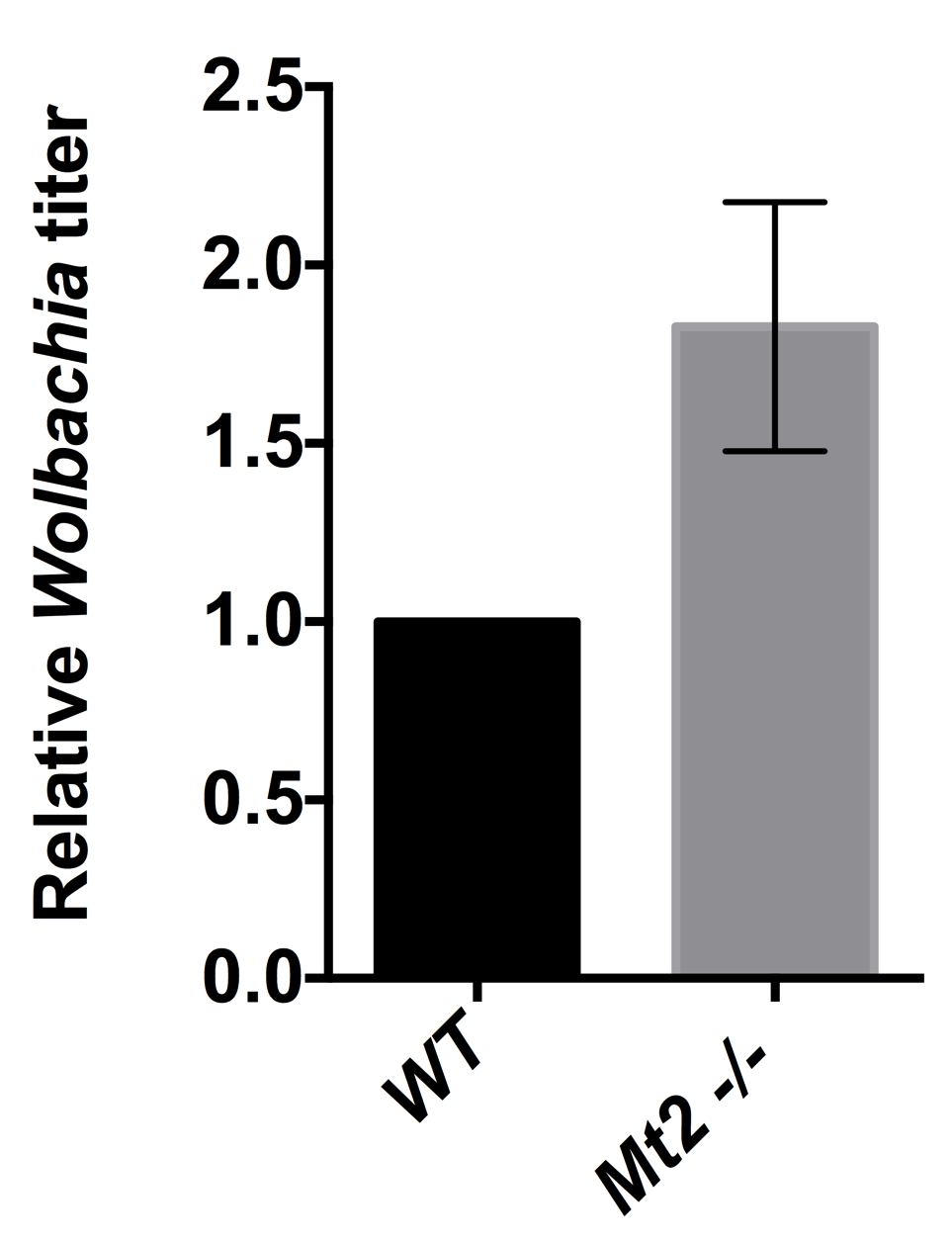

Supplement: S2 Fig — Relative Wolbachia titer in Mt2 -/- mutant flies was quantified using qPCR. Reported values are relative to isogenic wild-type flies (set at 1) and are represented as the mean of three independent biological replicates. All error bars represent standard error of mean (SEM). (TIFF) [file ppat.1006427.s002.tiff]

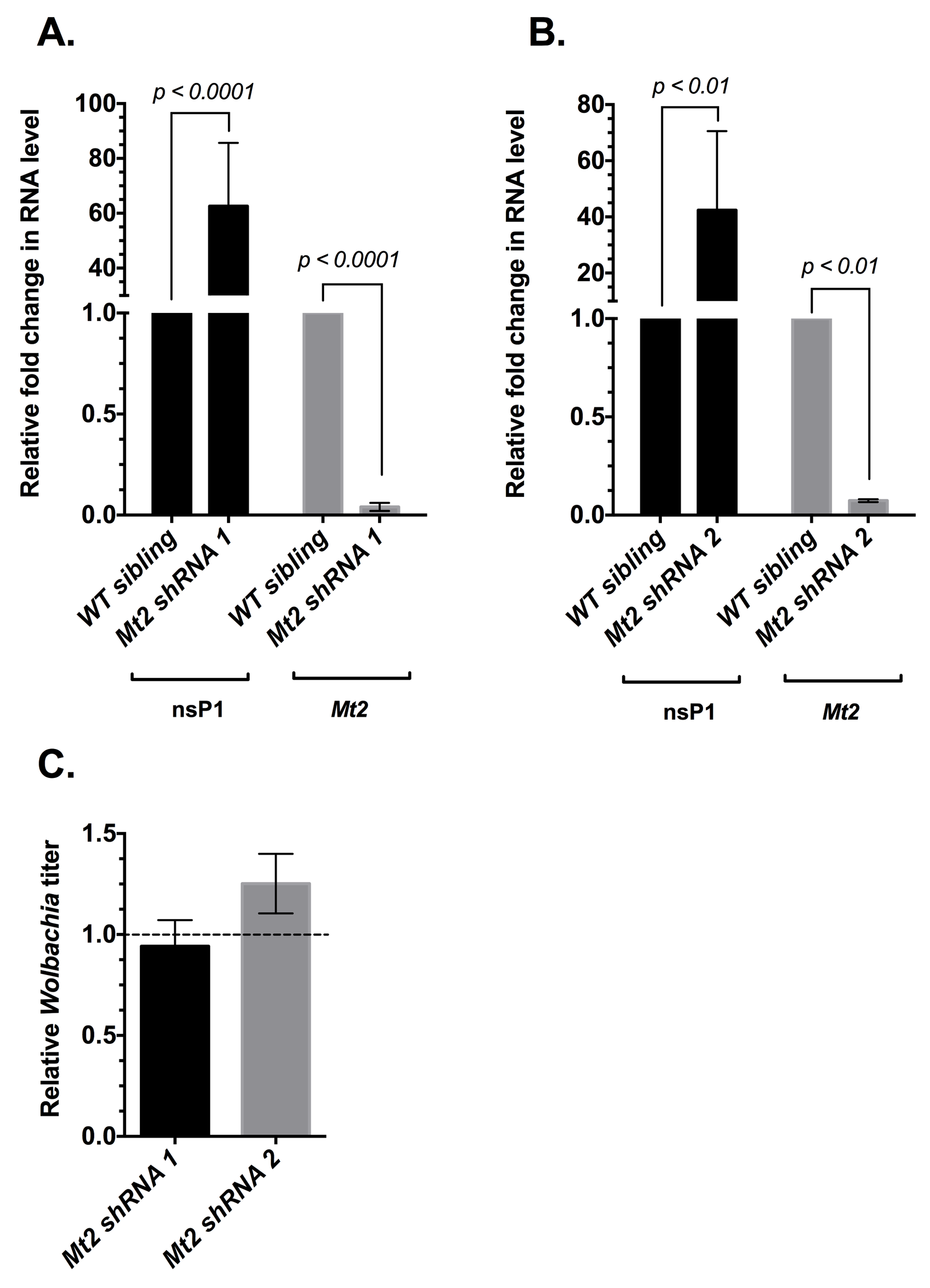

Supplement: S3 Fig — Mt2 expression was knocked down in Wolbachia infected transgenic RNAi fly stocks 38224 (Mt2 shRNA 1) and 42906 (Mt2 shRNA 2) by driving Mt2 shRNA expression via chromosome III Act5C-Gal4 driver (y1 w*; P{w[Act5C-GAL4}17bFO1/TM6B, Tb1) as described in Materials and Methods. For each set of crosses, siblings lacking the expression of Mt2 targeting shRNA were used as the wild-type controls. (A, B) Flies were challenged with SINV as described previously. Infection was allowed to last for 48 hours before RNA was extracted from whole fly tissues, followed by quantification of viral nsP1 and Mt2 expression via qRT-PCR. Quantitative analyses of gene expression in TRiP mutant flies was performed relative to their respective wild-type sibling controls (set at 1). Values represent the mean of six and four independent biological replicates, respectively. P values were calculated using Mann-Whitney U tests (Mt2 shRNA 1: nsP1 p < 0.0001, Mt2 p < 0.0001 and Mt2 shRNA 2: nsP1 p < 0.01, Mt2 p < 0.01). (C) Wolbachia titer was quantified using qPCR. Reported values are relative to respective wild-type sibling controls (set at 1) and are represented as the mean of three independent biological replicates. All error bars represent standard error of mean (SEM). (TIF) [file ppat.1006427.s003.tif]

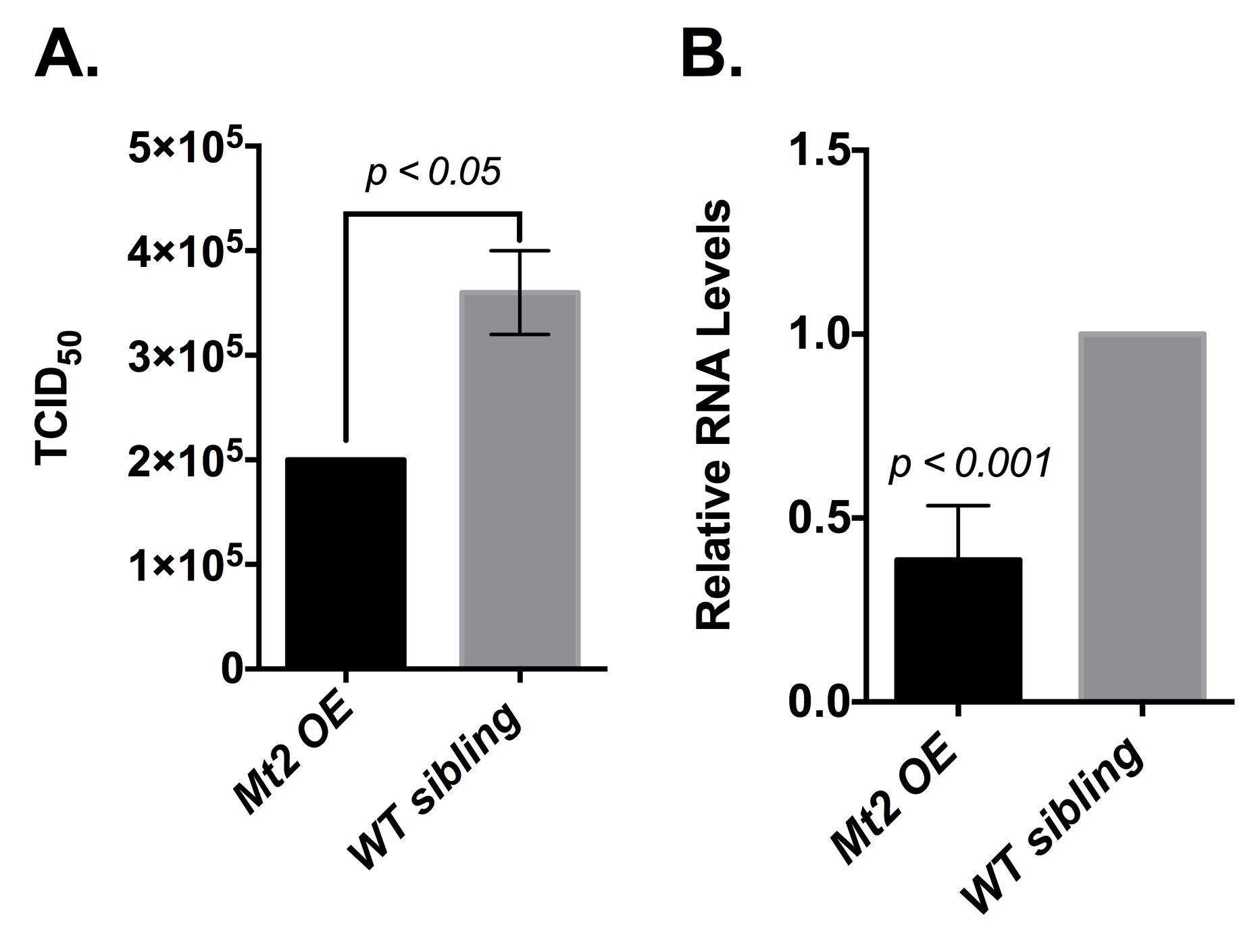

Supplement: S4 Fig — Wolbachia–free flies overexpressing Mt2 was generated by crossing uninfected UAS-Mt2 males with uninfected virgin Act5C-Gal4 driver females. Siblings generated through these crosses were used as wild-type controls. Flies were challenged with SINV that lasted for 48 hours before whole fly tissues were harvested. (A) Infectious virus was quantified as described previously, using end-point-dilution assay on BHK-21 cells. Values represent the mean of three independent biological replicates. P value was calculated using Mann-Whitney U test (p = 0.0476). (B) SINV RNA was quantified using qRT-PCR by probing against the viral E1 gene. Values represent the mean of three independent biological replicates. P value was calculated using Mann-Whitney U test (p < 0.001). All error bars represent standard error of mean (SEM). (TIFF) [file ppat.1006427.s004.tiff]

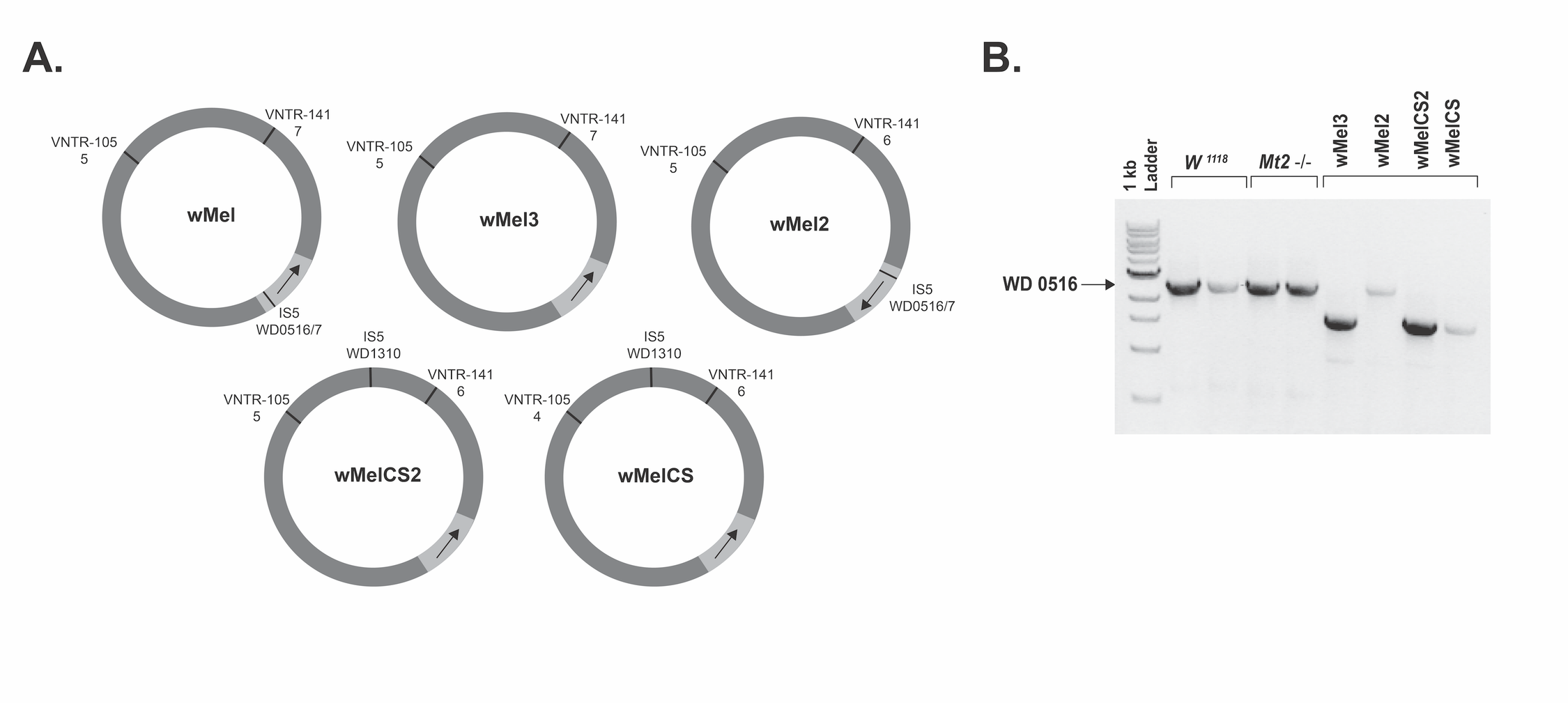

Supplement: S5 Fig — PCR based genotypic analyses was carried out to identify native D. melanogaster Wolbachia strain(s) present in the wild-type (W1118) and Mt2 -/- flies used in this study. (A) Genomic maps of common Wolbachia strains present in D. melanogaster indicate the location of distinct chromosomal IS5 transposon elements present or absent within either WD1310 or WD0516/7 loci. (B) Genomic region spanning the WD0516 loci was amplified using DNA isolated from W1118 and Mt2 -/- fly lysates (n = 3/sample) (Left). Controls obtained from flies infected with Wolbachia strains either wMel3, wMel2, wMelCS2 or wMelCS (Right). Increased shift in band migration indicate the presence of IS5 element at the WD0516 locus. (TIF) [file ppat.1006427.s005.tif]
