# Supplementary material for: Wolbachia elevates host methyltransferase expression to block an RNA virus early during infection
Source: PLoS Pathog. 2017 Jun 15;13(6):e1006427. doi: 10.1371/journal.ppat.1006427 (PMC5472326; doi:10.1371/journal.ppat.1006427)
Supplement: S1 Table — (PDF) [file ppat.1006427.s006.pdf]

**S1 Table. Primer sets used in this study for qRT-PCR and PCR* analyses.**

| | Target Gene | Forward Primer Sequence (5' – 3') | Reverse Primer Sequence (5' – 3') |
|---|---|---|---|
| **1** | Sindbis virus non-structural protein 1 (*nsP1*) | AAGGATCTCCGGACCGTA | AACATGAACTGGGTGTCGAAG |
| **2** | Sindbis virus glycoprotein E1 | TCAGATGCACCACTGGTCTCAACA | ATTGACCTTCGCGGTCGGATACAT |
| **3** | *18S (D. melanogaster)* | CGAAAGTTAGAGGTTCGAAGGCGA | CCGTGTTGAGTCAAATTAAGCCGC |
| **4** | *Wolbachia surface protein (wsp)* | CATTGGTGTTGGTGTTGGTG | ACCGAAATAACGAGCTCCAG |
| **5** | *DNA/RNA methyltransferase 2 (Mt2)* | CCGTGGCGTGAAATAGCG | ACACCGCTTTCGGAGGACG |
| **6** | *Vago* | CAGCCAAGCGATTCCTTATC | CGACCCGTCAATGTATCCATAC |
| **7** | *Attacin C (attC)* | GCAGAATCAGCTTGCCA | GCCCACCCAGCTCCAA |
| **8** | *Drosomycin (droso)* | CATTTACCAAGCTCCGTGAGAACC | GATTTAGCATCCTTCGCACCAGCA |
| **9** | *Diptericin B (dptB)* | TTATCCCTATCCTGATCCCCG | AGATCGAATCCTTGCTTTGGG |
| **10** | *Virus induced RNA-1 (vir-1)* | GATCCCAATTTTCCCATCAA | GATTACAGCTGGGTGCACAA |
| **11** | *T7 dsRNA** | GAATTAATACGACTCACTATAGTATTTCGGGTCTTAGAACTATCAAGAGTAGTTCTAAGA | GAATTAATACGACTCACTATAGTATTTCGGGTCTTAGAACTACTCTTGA |
| **12** | *IS5-WD0561* | CCATCAAGGTCTCTTTCA | TGCAAGGAAAACTAAACCAG |
